# Developing an *in vitro* screening assay platform for evaluation of antifibrotic drugs using precision-cut liver slices

**DOI:** 10.1186/s13069-014-0017-2

**Published:** 2014-12-16

**Authors:** Satish Kumar Sadasivan, Nethra Siddaraju, Khaiser Mehdi Khan, Balamuralikrishna Vasamsetti, Nimisha R Kumar, Vibha Haridas, Madhusudhan B Reddy, Somesh Baggavalli, Anup M Oommen, Raghavendra Pralhada Rao

**Affiliations:** Connexios life sciences private limited, No-49, Shilpa vidya, 1st Main, 3rd phase, J P nagara, Bangalore, 560078 India

**Keywords:** Liver slice, Fibrosis, Screening platform, Myofibroblast, Stellate cells

## Abstract

**Background:**

Precision-cut liver slices present different cell types of liver in a physiological context, and they have been explored as effective *in vitro* model systems to study liver fibrosis. Inducing fibrosis in the liver slices using toxicants like carbon tetrachloride is of less relevance to human disease conditions. Our aim for this study was to establish physiologically relevant conditions *in vitro* to induce fibrotic phenotypes in the liver slices.

**Results:**

Precision-cut liver slices of 150 μm thickness were obtained from female C57BL/6 J mice. The slices were cultured for 24 hours in media containing a cocktail of 10 nM each of TGF-β, PDGF, 5 μM each of lysophosphatidic acid and sphingosine 1 phosphate and 0.2 μg/ml of lipopolysaccharide along with 500 μM of palmitate and were analyzed for triglyceride accumulation, stress and inflammation, myofibroblast activation and extracellular matrix (ECM) accumulation. Incubation with the cocktail resulted in increased triglyceride accumulation, a hallmark of steatosis. The levels of *Acta2*, a hallmark of myofibroblast activation and the levels of inflammatory genes (IL-6, TNF-α and C-reactive protein) were significantly elevated. In addition, this treatment resulted in increased levels of ECM markers - collagen, lumican and fibronectin.

**Conclusions:**

This study reports the experimental conditions required to induce fibrosis associated with steatohepatitis using physiologically relevant inducers. The system presented here captures various aspects of the fibrosis process like steatosis, inflammation, stellate cell activation and ECM accumulation and serves as a platform to study the liver fibrosis *in vitro* and to screen small molecules for their antifibrotic activity.

## Background

Liver fibrosis is a pathological condition that results due to progressive accumulation of extracellular matrix in the liver. Several etiological factors like viral infection, alcohol abuse, insulin resistance and metabolic disorder contribute to the development of fibrotic phenotype [1]. It is a complex process involving various cell types of liver including hepatocytes, several immune cell types and stellate cells [2,3]. Following an initial injury to the liver (mainly to hepatocytes), the hepatic stellate cells get activated and differentiate into myofibroblasts, acquiring a pro-inflammatory and fibrogenic properties [4], and this event coupled with several other dysregulations leads to excess production of extracellular matrix (ECM). Uncontrolled liver fibrosis can eventually lead to total liver failure and it is one of the top 10 causes of mortality in the western world [5]. An effective cure for liver fibrosis is not available yet, and part of the reason for the slow progress of the pharmaceutical industry in this direction is lack of an effective *in vitro* model system to screen the small molecules [6,7]. Several research groups are working toward mechanisms underlying the development of disease and to identify potential antifibrotic compounds. The success of these studies would greatly depend on employing a suitable model system that captures various aspects of liver fibrosis as motioned above. Cell lines and isolated primary cultures serve as good model systems to address mechanism-based questions and to understand the cell type-specific biology. However, they fail to represent the liver as a multicellular system in which several cell types and cell-cell interactions contribute toward fibrogenesis [5]. Precision-cut liver slices have recently been evaluated for their use in studies with liver fibrosis [8-10], and they are more promising as model systems when compared to cell line-based systems. One major advantage of employing them as a model system is that they present several cell types of liver in a physiological milieu and they retain crucial interactions between different cell types and between cells and their ECM.

Earlier studies have used carbon tetrachloride (CCL_4_) as an inducer of liver fibrosis in a liver slice model system. CCL_4_ captures several endpoints involved in liver fibrosis, and is one of the oldest toxins known to stimulate fibrotic phenotype in the liver. However CCL_4_ is a nonphysiological challenge, and it has no etiological significance in human disease [11] but only leads to biochemical and histological changes similar to those of human disease condition [12]. Liver slices prepared from the rats with established fibrosis is a more physiologically relevant model, and this system has been used for screening antifibrotic compounds [8,13]. However, developing this model system can be time consuming, requiring about 3 to 4 weeks for the animals to develop disease.

In the present study, we report on developing liver fibrosis in liver slices using physiological signals that will activate key signaling pathways effectively and finally result in important end points relevant to NAFLD/fibrosis - triglyceride accumulation, hepatocyte dysfunction and inflammation, hepatic stellate cell activation, and ECM remodeling with increased collagen production.

## Results and discussion

Several signaling pathways are activated during pathogenesis of fibrosis, and each of these pathways contributes at various stages of the pathology finally leading to hepatic stellate cell activation and ECM production. The key pathways that contribute can be broadly categorized into inflammatory pathway, growth factor signaling and lipid signaling pathway. Most important among these pathways are the inflammatory pathway and the growth factor signaling mediated by TGF-β and PDGF signaling [2,10].

TGF-β is one of the potent inducers of fibrogenesis [14]. It plays a major role in the transformation of hepatic stellate cells into myofibroblasts and stimulates the synthesis of extracellular matrix proteins while inhibiting their degradation [15]. TGF-β signaling pathways have been explored as a target for fibrosis therapy [16]. PDGF is another potent proliferative factor for hepatic stellate cells and myofibroblasts during liver fibrogenesis [17]. During the process of fibrogenesis, it is secreted by a variety of cell types such as hepatocytes, kupffer cells and activated hepatic stellate cells, and many pro-inflammatory cytokines mediate their mitogenic effects via the autocrine release of PDGF [17].

Sphingosine 1 phosphate is well known for its diverse biological roles [18]. In the context of tissue fibrosis, S1P influences various aspects of fibroblast migration, stellate cell activation, myofibroblast differentiation and vascular permeability [19]. Several studies have established a causal connection between S1P and fibrosis of various organs like liver, lung and heart [20-22].

Phospholipid growth factors like lysophosphatidic acid (LPA) are known for their growth factor-like activity [23,24]. LPA exerts its action through well-characterized membrane receptors and has been found to promote cell division and migration and to inhibit apoptosis [25]. Relevant to fibrosis, LPA is shown to facilitate myofibroblast differentiation and ECM generation through activation of Rho-ROCK pathway [26,27].

Lipopolysaccharides (LPS), the cell wall derivatives of gram negative bacteria, activate toll-like receptor (TLR) pathways. The TLRs are expressed on variety of liver cell types that are central to the process of fibrosis like hepatocytes, kupffer cells and HSCs [28]. TLR pathways play critical role in fibrogenesis [28,29].

**Figure 1:**
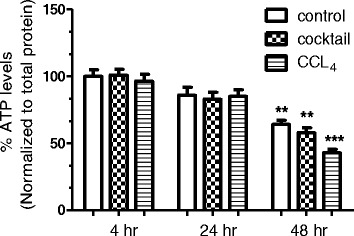
**Viability of liver slices.** Liver slices of 150 μm thickness were obtained and incubated with Williams E media supplemented with 15% fetal bovine serum and 1% GlutaMAX for indicated time periods. ATP levels were estimated at the end of the experiment, and levels were normalized to total protein content. The ATP levels in the control slices (incubated for 4 hours) were normalized to 100 and the percent ATP levels were calculated accordingly. **P <0.01 and ***P <0.001, when compared to respective samples at 4 hours.

In order to activate the signaling pathways discussed above, we formulated a cocktail containing 10nM each of TGF-β, PDGF, 5 μM each of lysophosphatidic acid and sphingosine 1 phosphate, and 0.2 μg/ml of lipopolysaccharide, along with 500 μM of palmitate. Palmitate was incorporated in the cocktail to facilitate lipid accumulation in the liver slices. We refer to this cocktail as IGL cocktail (denoting inflammatory, growth factor and lipid mediator). Viability of liver slices was estimated as a measure of total ATP content of the slices. Liver slices retained significant viability during the treatments for up to 24 hours as indicated in Figure 1. However, when extended up to 48 hours, the viability of the control and the cocktail treated slices declined to about 64% and 60% of the initial value, respectively. Most notably, the CCL_4_ treatment for 48 hours resulted in a drastic reduction in viability, with the treated slices showing only about 43% viability. This might be due to the more pronounced toxic effects of CCL_4,_ when compared to those of the IGL cocktail.

### An inflammatory, growth factor and lipid mediator cocktail (IGL) system captures the aspects of steatosis and inflammation

**Figure 2:**
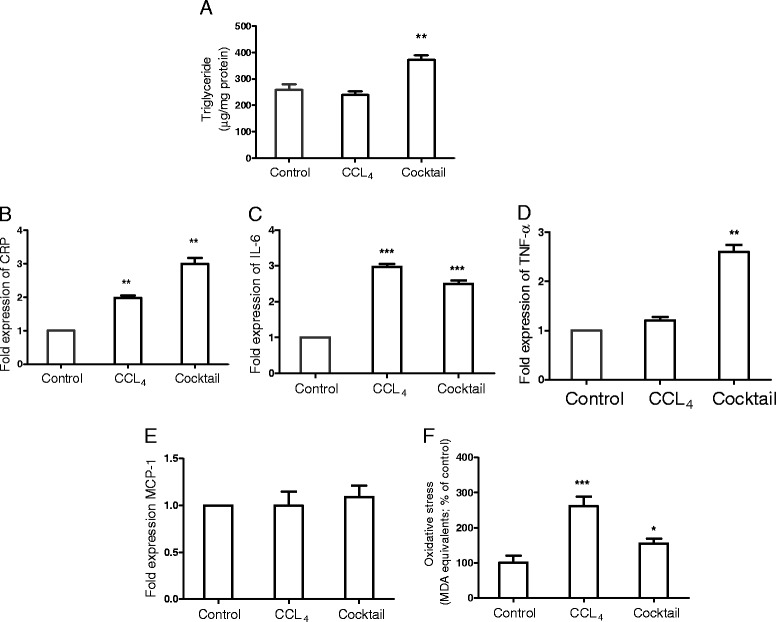
**Steatosis, inflammation and oxidative stress in the liver slices cultured in the inflammatory, growth factor and lipid mediator (IGL) cocktail.** Liver slices of 150-μm thickness were cultured either with CCL_4_ or IGL cocktail for 24 hours, after which triglyceride levels **(A)** and oxidative stress levels **(F)** were estimated. RNA was isolated from the liver slices, and expression levels of CRP **(B)**, IL-6 **(C)**, TNF-α **(D)** and MCP-1 **(E)** were quantified by real-time quantitative PCR using the beta actin gene as the endogenous control. Expression levels of each of the genes in the control samples (liver slices cultured with culture media alone without CCL_4_ or IGL cocktail) were normalized to 1. Values represent mean ± SEM (n = 4 per group). An unpaired student t-test was used for statistical comparison. *P <0.05, **P <0.01, and ***P <0.001, when compared to control.

Development of liver fibrosis associated with NASH (nonalcoholic steatohepatitis) can be explained by two hits theory. The ‘first hit’ is marked by the accumulation of lipids in hepatocytes while the ‘second hit’ leads to hepatocyte injury, inflammation and fibrosis [30,31]. Contrary to the initial belief that fat accumulation in the liver is a benign condition, several studies have established hepatic fat storage as a risk factor for the progression of hepatic fibrosis [32]. Triglyceride (TG) accumulation and lipid droplet formation tightly correlate with pathophysiological mechanisms in NASH [33] and TG accumulation is a potent trigger for hepatocytes injury and inflammation. In our assay system we tested the levels of triglycerides. When treated with IGL cocktail, the liver slices showed increased triglyceride accumulation (Figure 2A). CCL_4_ treatment under similar conditions did not result in any change in the triglyceride levels.

Increased inflammation in these slices was evident with increased expression levels of CRP, IL6 and TNF-α (Figure 2B, C and D) following treatment with the IGL cocktail. CRP is a well-known marker of inflammation and is also proposed as a marker of nonalcoholic fatty liver disease [34,35]. Animal model and clinical studies indicate that TNF-α is involved in mediating both initial and advanced stages of liver damage [36]. IL-6 is a pleiotropic inflammatory cytokine and is involved synthesis of broad spectrum of acute phase proteins, chronic inflammation and fibrogenesis [37]. Monocyte chemoattractant protein 1 (MCP-1) plays an important an role in inflammation, liver injury and NASH [38,39] and is used as a reliable marker for inflammation. However, in our study, MCP-1 levels did not significantly increase following treatment, either with the IGL cocktail or CCL_4_ (Figure 2E). The reason behind this could be kinetics of expression of MCP-1 during the process of *in vitro* fibrosis (as in the current study), and we speculate that MCP-1 expression is a very early event during fibrosis. This is further supported by a study that reports that following CCL_4_ treatment rat liver shows increased expression of MCP-1 between 6 to 48 hours, but is not detectable after 60 hours [38]. Oxidative stress is known to significantly contribute to fibrogenesis, and reactive oxygen species and the lipid peroxides are shown to enhance inflammation and cellular damage, stellate activation and production of collagen [40-42]. To assess if the IGL cocktail treatment influences the oxidative stress in the slices, we estimated the oxidative stress in the liver slices. As shown in the Figure 2F, the IGL cocktail treatment resulted in about a 50% increase in oxidative stress levels. CCL_4_ treatment, in comparison, had higher levels of oxidative stress compared to the cocktail treatment.

### Inflammatory, growth factor and lipid mediator cocktail treatment results in stellate cell activation

**Figure 3:**
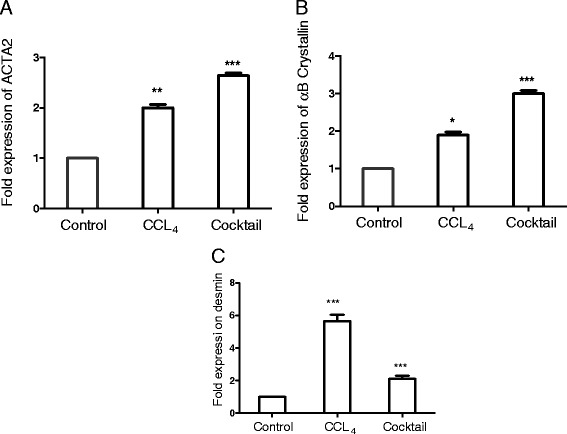
**Effect of the inflammatory, growth factor and lipid mediator (IGL) treatment on the hepatic stellate cell activation.** Liver slices of 150-μm thickness were cultured either with CCL_4_ or IGL cocktail for 24 hours and expression levels of *Acta2* **(A)**, αB-crystallin **(B)** and desmin **(C)** were quantitated by real-time quantitative PCR using the beta actin gene as an endogenous control. Expression levels of each of the genes in the control samples (liver slices cultured with culture media alone without CCL_4_ or IGL cocktail) were normalized to 1. Values represent mean ± SEM (n = 4 per group). An unpaired student t-test was used for statistical comparison. *P <0.05, **P <0.01, and ***P <0.001, when compared to control.

Stellate cell activation is a key event in liver fibrosis, and it involves the process of transition of a quiescent, adipose-like vitamin-A storing cell to a highly fibrogenic cell [41]. Upon activation, stellate cells undergo a programmed cascade of events to differentiate into myofibroblasts. Myofibroblasts are more motile and contractile in nature, and this functional transition is paralleled with an increased expression of *Acta2* [43,44]. We assessed the expression of this gene following exposure to IGL cocktail. As shown in the Figure 3A, with IGL treatment, the levels of *Acta2* were increased appreciably. αB-crystallin is small heat shock protein belonging to the HSP20 family, and it is known to protect the cells against protein degradation. It is implicated as a marker for early hepatic stellate cell activation [45]. Following treatment with IGL cocktail the levels of αB-crystallin were found to be upregulated in the liver slices (Figure 3B). Desmin is an intermediate filament typical of contractile cells and is used as a gold standard for stellate cell activation [9,46]. Upon stimulation by IGL cocktail, the levels of desmin increased in the liver slices as indicated in Figure 3C

.

### Inflammatory, growth factor and lipid mediator cocktail system captures the aspects of extracellular matrix accumulation and remodeling

**Figure 4:**
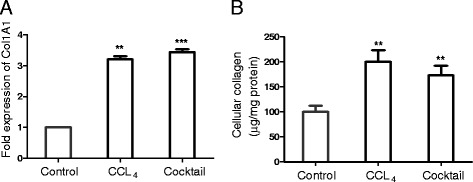
**Effect of inflammatory, growth factor and lipid mediator (IGL) treatment on collagen levels.** Liver slices of 150-μm thickness were cultured either with CCL_4_ or IGL cocktail for 24 hours, and the expression levels of collagen was quantitated by real-time quantitative PCR using the beta actin gene as an endogenous control **(A)**. Expression levels of each of the genes in the control samples (liver slices cultured with culture media alone, without CCL_4_ or IGL cocktail) were normalized to 1. Total collagen in the liver slices was estimated **(B)** using Sirius red dye. Values represent mean ± SEM (n = 4 per group). An unpaired student t-test was used for statistical comparison. **P <0.01 and ***P <0.001, when compared to control.

**Figure 5:**
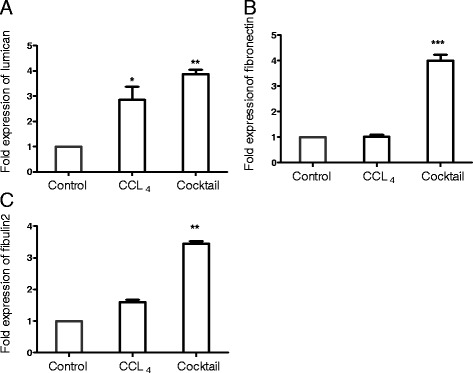
**Effect of inflammatory, growth factor and lipid mediator treatment on extracellular matrix (ECM) accumulation.** Liver slices of 150 μm thickness were cultured either with CCL_4_ or IGL cocktail for 24 hours and the expression levels of Lumican **(A)**, Fibronectin **(B)** and Fibulin2 **(C)** were quantitated by real-time quantitative PCR using the beta actin gene as an endogenous control. Expression levels of each of the genes in the control samples (liver slices cultured with culture media alone without CCL_4_ or IGL cocktail) were normalized to 1. Values represent mean ± SEM (n = 4 per group). An unpaired student t-test was used for statistical comparison. *P <0.05, **P <0.01, and ***P <0.001, when compared to control.

An imbalance between ECM synthesis and degradation leads to excessive ECM accumulation, an end point that defines liver fibrosis. To evaluate if the treatment with the IGL cocktail resulted in fibrogenesis, collagen content was estimated in the liver slices. Collagen is a major protein constituent of the ECM. As indicated in Figure 4A and B, the collagen content of the liver slices was significantly increased following treatment with the IGL cocktail. In addition to collagen, the levels of other ECM proteins - fibulin2, lumican and fibronectin - increased in response to treatment with the IGL cocktail (Figure 5).

**Figure 6:**
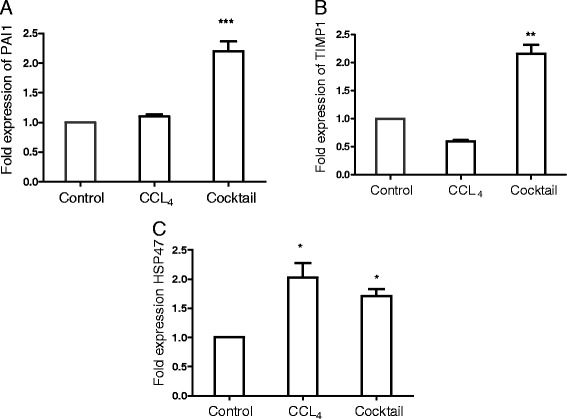
**Effect of inflammatory, growth factor and lipid mediator (IGL) treatment on extracellular matrix (ECM) remodeling.** Liver slices of 150-μm thickness were cultured either with CCL_4_ or IGL cocktail for 24 hours and expression levels of PAI-1**(A)**, TIMP1**(B)** and HSP47 **(C)** were quantitated by real-time quantitative PCR using beta actin gene as endogenous control. Expression levels of each of the genes in the control samples (liver slices cultured with culture media alone without CCL_4_ or IGL cocktail) were normalized to 1. Values represent mean ± SEM (n = 4 per group). An unpaired student t-test was used for statistical comparison *P <0.05, **P <0.01, and ***P <0.001, when compared to control.

PAI-1 is an inhibitor of serine protease tissue plasminogen activator (tPA) and urokinase (uPA) and is a potent inhibitor of fibrinolytic activity. Increased levels of PAI-1 are correlated with fibrogenesis [47]. While increased synthesis of collagen contributes to ECM accumulation, inhibition of uPA and tPA resulting from elevated PAI-1 sustains the fibrosis [48]. Increases in the PAI-1 levels were seen upon treatment with IGL cocktail (Figure 6A). In addition to PAI-1, studies have identified that TIMPs (tissue inhibitor of metalloproteinases) play a key role in the fibrosis and a correlation between TIMP levels, and fibrosis has been established in a rat model of liver fibrosis [49]. As indicated in Figure 6B, TIMP1 levels were significantly increased in response to treatment with the cocktail. CCL_4_ treatment, however, did not result in appreciable changes in the levels of either PAI-1 or TIMP1. HSP47 is a heat-shock protein expressed mainly by the myofibroblasts, and it acts as a molecular chaperone for procollagen molecules. This function of HSP47 results in stabilization of collagen molecule, an important ECM protein whose levels are increased in the fibrosis. The level of HSP47 has been shown to be upregulated in the animal models of liver fibrosis [50]. Hence, we estimated the expression levels of HSP47 following incubation with the cocktail. The levels of HSP47 were increased following treatment with the cocktail as indicated in Figure 6C.

## Conclusions

Our assay system indeed captures critical aspects of the pathology-like inflammation and oxidative stress, hepatic stellate cell activation and extracellular matrix overproduction. Although this cocktail is not exhaustive in representing all the signaling pathways, it nevertheless corresponds to diverse arms of signaling networks involved in fibrogenesis. The fact that the IGL cocktail treatment results in a steatotic phenotype in the slices as measured in terms of triglyceride accumulation makes it very suitable for use in studying fibrosis in the background of steatosis. It should be noted, however, that this system does not represent progression of fibrosis pathology from steatosis to steatohepatitis and fibrogenesis, in which case one would expect development of fibrogenesis in the slices following incubation with palmitic acid alone. We feel that this would not be practically possible in a liver slice system given that progression from steatosis to fibrosis requires a long time, at least *in vivo* [51], and translating this in an *ex vivo* set-up such as liver slice may be limited due to viability issues. Nevertheless, triglyceride accumulation in the slices sets up a suitable background of steatosis that contributes to key aspects of liver fibrosis.

## Methods

### Materials

The William’s E Media, GlutaMAX, fetal bovine serum for the cell culture, ATP estimation kit, recombinant TGF-β and the PDGF were purchased from Life Technologies USA. Lysophosphatidic acid, lipopoly saccharide and palmitic acid were purchased from Sigma Aldrich. The cDNA synthesis kit was from BioRad, the qPCR kit was from KAPA Biosystems, and the triglyceride estimation kit (TAG reagent) was from Diasys.

### Animals

C57BL/6 J female mice were housed at 22 ± 3°C, with a relative humidity of 50 to 70% on a 12 h light and 12 h dark cycle with artificial fluorescent tubes. Animals were fed *ad libitum* with normal chow diet. Mice aged between 8 to 12 weeks were used for preparation of liver slices. In order to minimize any possible variations emanating from sex differences, only female mice were used throughout the study. The study protocol, animal maintenance, and experimental procedures were all approved by the Institutional Animal Ethics Committee (IAEC) of Connexios Life Sciences, which is approved by CPCSEA (Committee for the Purpose of Control and Supervision of Experiments on Animals, government of India).

### Preparation of liver slices

Williams E media was prepared with 15% FBS and 1% GlutaMAX. Five milliliters of media was dispensed to each T25 flask. 8 to 12 week old C57BL/6 J animals were euthanized using isoflurane, and the liver was collected in a Petri dish containing pre-warmed Williams E media The lobes of the liver were separated and were cut into small pieces of about 10 mm^3^. Precision-cut liver slices of 150 μm thickness was obtained using automated vibrating blade microtome (Leica VT 1200S), and the slices were collected under aseptic conditions into pre-warmed media. About 8 to 10 precision-cut liver sections were then distributed to each T25 flask on a random basis. The thickness of the liver slices influences the viability of the cells and oxygen diffusion during incubations [5]. Using the slices of greater thickness would result in reduced oxygen diffusion into the slices, while using slices of lesser thickness can affect the viability of the cells in the outer layer of the slices. In literature people have successfully used thicknesses as low as 100 μm [52] and also the slices up to about 250 μm [5]. In the current study, we use slices of 150-μm thickness, and this thickness was good enough to retain viability of the slices for up to 24 hours as discussed in results section.

### Liver slice culture

Liver slices from mouse (8- to 12-week-old C57BL/6 J) were cultured in William’s E Media supplemented with 15% Fetal Bovine serum (FBS) and 1% GlutaMAX [10]. Cultures were maintained in a humidified atmosphere of 95% air and 5% CO_2_ at 37°C. In order to induce a fibrotic phenotype, the slices were cultured for 24 hours in the media with a cocktail containing 10nM each of TGF-β, PDGF, 5 μM each of lysophosphatidic acid and sphingosine 1 phosphate and 0.2 μg/ml of lipopolysaccharide. Where mentioned, CCL_4_ was used at a concentration of 0.1%.

### Quantitative real-time PCR

Total RNA was isolated from each liver slice using TRIZOL (Ambion), and 1 μg of RNA was reverse-transcribed with the iScript cDNA synthesis kit (BIO RAD). The qRT-PCR assays were performed in 10-μl reactions containing 1× SYBR Green Master Mix buffer (KAPA), and 300 nM gene-specific primers. Assays were performed using a CFX96 Real-Time System (Bio-Rad Laboratories). Samples were incubated in SYBR Green Master Mix for an initial denaturation at 95°C for 3 min, after which 40 PCR cycles were performed, with each cycle consisting of 95°C for 10 s, 60°C for 10 s and 72°C for 15 s. Amplification of specific transcripts was confirmed by melting curve profiles (cooling the sample to 68°C and heating slowly to 95°C with measurements of fluorescence) at the end of each PCR. Each gene expression was calculated relative to beta actin gene, which was used as an internal control by using the ΔΔCT analysis method. The primer sequences are listed in Table 1.

**Table 1:** **Sequences of the primers used in this study**

**Gene**	**Primer**
CRP	Forward TGG TGG GAG ACA TCG GAG AT
	Reverse GCC CGC CAG TTC AAA ACA TT
IL6	Forward CTG ATG CTG GTG ACA ACC AC
	Reverse CAG AAT TGC CAT TGC ACA AC
TNF-α	Forward TAG CCA GGA GGG AGA ACA GAA A
	Reverse CCA GTG AGT GAA AGG GAC AGA A
ACTA2	Forward GCCAGTCGCTGTCAGGAACCC
	Reverse: GCGAAGCCGGCCTTACAGAGC
αB-Crystallin	Forward TTC TTC GGA GAG CAC CTG TT
	Reverse CCC CAG AAC CTT GAC TTT GA
Collagen (Col1a1)	Forward ATG GCC AAC CTG GTG CGA AAG G
	Reverse ACC AAC GTTA CCA ATG GGG CCG
Lumican	Forward TGC AGT GGC TCA TTC TTG AC
	Reverse GGA CTC GGT CAG GTT GTT GT
Fibulin 2	Forward GAA CTT CTC GGA TGC TGA GG
	Reverse CAA CTG GCC AGG GTG TTA CT
PAI-1	Forward CAG CCC TTG CTT GCC TCA T
	Reverse CCG AGG ACA CGC CAT AGG
MCP-1	Forward AGC ACC AGC CAA CTC TCA CT
	Reverse TCA TTG GGA TCA TCT TGC TG
HSP47	Forward GTT TCT TGG GAC AGG CAG GAG
	Reverse GCC TGC CTT TTT CAT TCT GGG C
Desmin	Forward TCG CGG CTA AGA ACA TCT CT
	Reverse TCG GTA TTC CAT CAT CTC CTG

### Triglyceride estimation

Liver slices were bead lysed in 100 μl lysis buffer ( 50 mM Tris, 150 mM NaCl, 0.1% Triton X 100, pH 7.4), at 25 Hz for 5 minutes. The lysed samples were centrifuged at 10,000 rpm for 10 minutes, and the supernatant was taken for analysis. Next, 200 μl of TAG reagent (Triacyl glycerol reagent, supplied with the kit) was added to 10 μl of the sample or standard and incubated at 37°C for 10 min and absorbance was read at 500 nm. The TAG was normalized to total cellular protein.

### Soluble collagen estimation

Liver slices were bead lysed in 100 μl lysis buffer ( 50 mM Tris, 150 mM NaCl, 0.1% Triton X 100, pH 7.4) at 25 Hz for 5 minutes. The lysed samples were centrifuged at 10,000 rpm for 10 minutes and the supernatant was taken for analysis. Next, 200 μl of Sirius red dye was added to 40 μl of sample and incubated at room temperature for 2 h. The samples were centrifuged at 12,000 rpm for 15 minutes. The pellet was washed with 500 μl of phosphate buffered saline (PBS) and then with 500 μl of 0.05 N hydrochloric acid. Pellet was dissolved in 100 μl of 0.2 N Sodium hydroxide and absorbance read at 540 nm. The collagen levels were normalized to total cellular protein content.

### Assay for viability

Immediately following termination of the experiment, liver slices were lysed in 100 μl lysis buffer (0.1 N NaOH, 0.1% Triton X100). The samples were centrifuged at 10,000 rpm for 10 minutes, and the supernatant was used for estimation of ATP using ATP determination kit following manufacturer’s instructions (Life Technologies).

## Abbreviations

ECM: extracellular matrix
IL-6: interleukin-6
PDGF: platelet derived growth factor
TAG: triacyl glycerol
TG: triglcyerides
TGF-β: transforming growth factor beta
TNF-α: tumor necrosis factor alpha
